# Spatial Processing Is Frequency Specific in Auditory Cortex But Not in the Midbrain

**DOI:** 10.1523/JNEUROSCI.3034-16.2017

**Published:** 2017-07-05

**Authors:** Joseph Sollini, Robert Mill, Christian J. Sumner

**Affiliations:** Medical Research Council Institute of Hearing Research, University of Nottingham, Nottingham NG7 2RD, United Kingdom

**Keywords:** auditory cortex, frequency specificity, inferior colliculus, sound localization

## Abstract

The cochlea behaves like a bank of band-pass filters, segregating information into different frequency channels. Some aspects of perception reflect processing within individual channels, but others involve the integration of information across them. One instance of this is sound localization, which improves with increasing bandwidth. The processing of binaural cues for sound location has been studied extensively. However, although the advantage conferred by bandwidth is clear, we currently know little about how this additional information is combined to form our percept of space. We investigated the ability of cells in the auditory system of guinea pigs to compare interaural level differences (ILDs), a key localization cue, between tones of disparate frequencies in each ear. Cells in auditory cortex believed to be integral to ILD processing (excitatory from one ear, inhibitory from the other: EI cells) compare ILDs separately over restricted frequency ranges which are not consistent with their monaural tuning. In contrast, cells that are excitatory from both ears (EE cells) show no evidence of frequency-specific processing. Both cell types are explained by a model in which ILDs are computed within separate frequency channels and subsequently combined in a single cortical cell. Interestingly, ILD processing in all inferior colliculus cell types (EE and EI) is largely consistent with processing within single, matched-frequency channels from each ear. Our data suggest a clear constraint on the way that localization cues are integrated: cortical ILD tuning to broadband sounds is a composite of separate, frequency-specific, binaurally sensitive channels. This frequency-specific processing appears after the level of the midbrain.

**SIGNIFICANCE STATEMENT** For some sensory modalities (e.g., somatosensation, vision), the spatial arrangement of the outside world is inherited by the brain from the periphery. The auditory periphery is arranged spatially by frequency, not spatial location. Therefore, our auditory perception of location must be synthesized from physical cues in separate frequency channels. There are multiple cues (e.g., timing, level, spectral cues), but even single cues (e.g., level differences) are frequency dependent. The synthesis of location must account for this frequency dependence, but it is not known how this might occur. Here, we investigated how interaural-level differences are combined across frequency along the ascending auditory system. We found that the integration in auditory cortex preserves the independence of the different-level cues in different frequency regions.

## Introduction

Our perception of sounds in space relies on the integration of multiple physical cues. The dominant cue for high-frequency sound localization is the interaural level difference (ILD) (Rayleigh, 1907, Wightman and Kistler, 1992, Macpherson and Middlebrooks, 2002). ILD-based localization is enhanced by adding other localization cues (interaural timing differences, spectral cues) or by providing additional ILD information at a wider range of frequencies (Butler, 1986, Wightman and Kistler, 1992, Hartmann and Constan, 2002). Little is known about how additional frequency content is synthesized.

For a given spatial position in azimuth, the transfer function of the head imposes different ILDs at different frequencies (Feddersen et al., 1957, Shaw, 1974); ILD cues are frequency specific. If any across-frequency integration process led to ILD computation across broad frequency regions, this would “blur” the ILD cues and presumably reduce acuity. The situation would be worse for multiple sound sources, in which different frequency regions might contain energy from either (or both) sound sources. This leads to the prediction that across-frequency integration should preserve the frequency specificity of ILD cues. Consistent with this, psychophysical thresholds for detecting ILDs increase when there is a frequency mismatch between the ears (Francart and Wouters, 2007, Goupell et al., 2013).

Evidence suggests that ILD computation and across-frequency integration are partially segregated in the auditory pathway. ILD sensitivity is created in the lateral superior olive (LSO) (Galambos et al., 1959) and also the inferior colliculus (IC) (Adams and Mugnaini, 1984, Faingold et al., 1991, Smith, 1992, Yang and Pollak, 1994, 1998, Orton et al., 2016). Thereafter, there is little evidence of new interaural comparisons (Malmierca et al., 2005, Kyweriga et al., 2014). In contrast, cortical across-frequency integration is supported by extensive thalamocortical and intracortical connectivity (Kaur et al., 2005, Intskirveli et al., 2016). Therefore, the auditory pathway may first extract frequency-specific ILD cues and then integrate across frequency.

In a linear computation, order is not important and models of auditory cortex (AC) responses suggest that linear integration of spectral energy from each ear can predict spatial receptive fields (Schnupp et al., 2001, Mrsic-Flogel et al., 2005). Importantly, they also predict that the frequency specificity of ILD processing will be lost if a cell is integrating information from different frequency regions.

Currently, it is not clear how to reconcile physiological spatial receptive field properties, the architecture of ILD processing, and perception. Here, we tested the ability of the auditory system to make binaural comparisons across frequency. Using a stimulus design in which pure tones can differ in frequency, level or both between the ears, we sought to determine whether ILD sensitivity depends on the absolute frequencies of the tones in each ear, as predicted by linear models, or the difference in frequency across the ears. We show that neurons in primary AC integrate binaural information in a frequency-specific manner. In contrast, neurons in the IC are insensitive to spectral differences across the ears.

## Materials and Methods

### 

#### 

##### Animal preparation.

Subjects were tricolor pigmented guinea pigs (*Cavia porcellus*) of both sexes. Anesthesia was induced with an intraperitoneal injection of urethane (4.5 ml/kg of a 20% solution) supplemented with intramuscular injections of 0.2 ml of Hypnorm (fentanyl citrate 0.315 mg/ml, fluanisone 10 mg/ml) when necessary. Bronchial secretions were suppressed with a subcutaneous injection of 0.2 ml of atropine sulfate (600 mg/ml). Animals were tracheotomized and breathing and temperature were maintained artificially using a respirator and heating blanket (38°C). The soft tissue around the ear canal was surgically removed and the animal placed in a stereotaxic frame with hollow plastic specula for ear bars. The frame was located inside a sound-attenuating room. Pressure across the tympanic membrane was equalized bilaterally by sealing a small polythene tube into a small hole in the bulla. The foramen magnum was opened to release the pressure of the CSF.

For the cortical recordings, primary AC was exposed by making a craniotomy of about 7 mm^2^ (Wallace et al., 2000). For the IC recordings, a rectangular craniotomy was made on the top of the skull 10–14 mm behind bregma and 0–2.5 mm lateral from midline. This allowed access to the right IC from directly above through the overlying cerebral cortex (Palmer et al., 2013). In both cases, the dura was removed and the brain was covered with a layer of agar to promote recording stability and to prevent desiccation. All experiments were performed in accordance with UK Home Office regulations.

##### Neurophysiological recordings.

Neurophysiological recordings were conducted using in-house-manufactured multielectrode arrays (Bullock et al., 1988). Each multielectrode array held 1–6 glass-insulated tungsten electrodes (8–12 μm tip size to ensure good unit isolation) and was advanced into AC or IC with a piezoelectric motor (Burleigh Inchworm IW-700/710). Signals from the multielectrode array were band-pass filtered (0.16–6000 Hz) using a high-impedance headstage (Tucker-Davis Technologies RA16AC) and digitized using a preamplifier (Tucker-Davis Technologies RA16PA). This signal was then passed to a digital signal processor (RX7), where the signal was again filtered under the control of BrainWare (Tucker-Davis Technologies; 400–3000 Hz). Recordings were monitored online using BrainWare.

##### Stimulus presentation.

All stimuli were generated online using a Tucker-Davis Technologies RX6 and presented binaurally via a pair of custom-modified RadioShack 40–1377 tweeters (M. Ravicz, Eaton Peabody Laboratory). After surgery, the sound system was calibrated using a probe microphone close to the tympanic membrane to determine the frequency response of the presentation system. This was used to create flat spectrum output for subsequent sound presentation (±2dB, 0.1–25 kHz). Gaussian white noise was used as a search stimulus for cells (0.01–48 kHz, 100 ms duration, 2 ms cos^2^ on–off ramp, 70 dB SPL). For each cell, the pure tone frequency response area (FRA) for sounds presented to the contralateral ear was measured (100 ms duration, 6 ms cos^2^ on–off ramp, 1 s interstimulus interval, 20–80 dB SPL, 3–5 repeats of each frequency level combination). Contralateral FRAs were plotted in real time and used to determine the frequencies to be used for ILD presentation.

**Figure 1.**
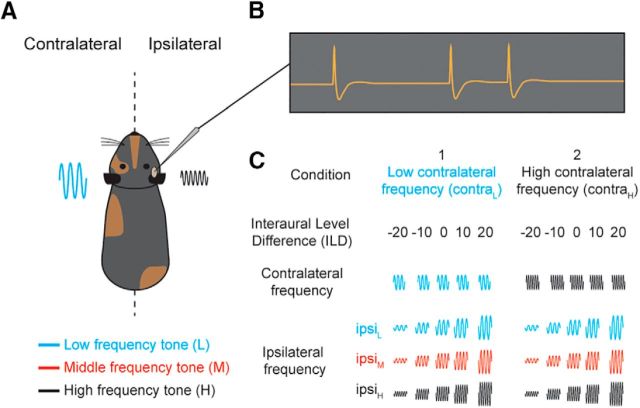
The ability of the auditory system to integrate spatial cues across frequency was tested in guinea pigs. ***A***, Experimental setup schematic, extracellular recordings were made in primary AC and the IC of anesthetized guinea pigs. Pure tones of various frequency and level combinations were presented simultaneously to both ears via in-ear inserts. Color indicates the relative frequency used. ***B***, Schematic neural trace. ***C***, Stimulus design. The contralateral tone level was held constant while the ipsilateral tone level was varied. For each contralateral tone frequency (high or low), three different ipsilateral tone frequencies were presented at a number of levels, creating ILDs across different frequency ranges.

For the binaural stimulus protocol, contralateral pure tones were selected to be evenly spaced in frequency (on an octave scale) and matched for excitability by varying sound level. Two frequencies within the contralateral FRA were selected, generally centered around characteristic frequency (CF), contra_L_ and contra_H_ (see Fig. 1*C*, left and right, respectively) with a frequency spacing of 0.25, 0.5, or 1 octave (see Fig. 3). To ensure that firing rates at the two frequencies were matched, a rate-level function was collected at each frequency and appropriate sound levels selected. ILD functions were measured using the “excitatory-monaural intensity” constant method in which contralateral tone sound levels were held constant while varying the ipsilateral sound level. Most ILDs ranged from −20 to +20 dB in 5 dB increments, which is well matched to the physiological range of ILDs for a guinea pig (Greene et al., 2014). Across-frequency binaural level sensitivity was tested by varying the interaural frequency difference (IFD). For each contralateral tone condition, three corresponding ipsilateral tone frequencies were used: one at each contralateral tone frequency (ipsi_L_ and ipsi_H_, respectively, one matched to a given contralateral tone, one mismatched) and one halfway in between on an octave spacing (ipsi_M_; see Fig. 1*C*). This meant that, for each contralateral tone condition, there were three IFDs. The same ipsilateral frequencies were used across the 2 tone contralateral conditions, making 2 × 3 = 6 ILD functions. Therefore, the IFDs were symmetrical and of the same frequency difference but of opposite sign for the two contralateral tone frequencies. Stimulus conditions were repeated between 10 and 50 times (usually >25 repeats) in AC and IC recordings. Across all the recordings, there were 5 possible IFD values in total: 0, 0.125, 0.25, 0.5, and 1 octave.

##### Data analysis.

Spike data were sorted offline using Plexon version 2.8.8 and processed in MATLAB (The MathWorks). Spikes within a window spanning the duration of the stimulus (0–100 ms) were used for all analyses. Monaural and binaural responses were calculated by counting the total number of spikes per presentation. To test for significant binaural interactions in each condition, the firing rates for each presentation were randomly resampled with replacement 500 times to produce a bootstrapped distribution of possible mean firing rates. This was then used to test for significant modulations of firing rate (*p* < 0.05) compared with the bootstrapped distribution of the response to the corresponding contralateral tone alone. ILD functions that did not modulate their firing rates significantly at one or more ipsilateral levels were not used for subsequent analysis.

**Figure 2.**
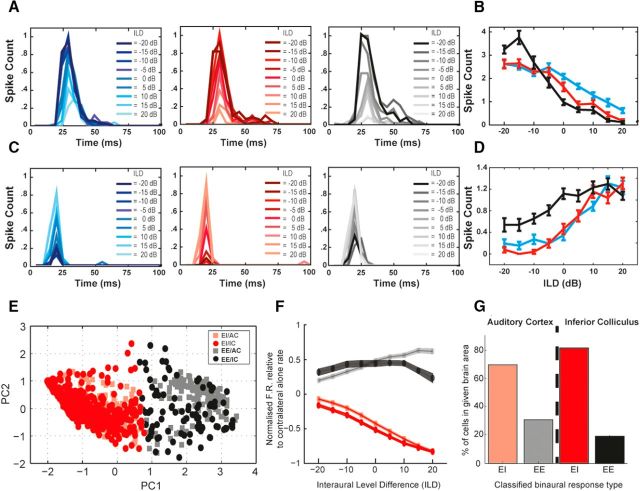
ILD functions with significant binaural interactions could be classified into two separate categories, inhibitory and facilitatory. ***A***, PSTHs from an example inhibitory unit in AC for a single contralateral tone condition and three different ipsilateral frequencies (left to right: ipsi_L_, ipsia_M_, ipsp_H_: blue, red, and black lines, respectively). Shading indicates ILD for each PSTH. ***B***, Number of spikes elicited by each stimulus in ***A*** with color indicating ipsilateral frequency. ***C*** and ***D*** show a corresponding example facilitatory unit. ***E***–***G***, Multiple ILD functions were collected for each cell and these were pooled together (for all cells) and PCA and clustering (agglomerative) performed. ***E***, First two principal components (PC1 and PC2) from the PCA. Clustering revealed two clusters across the two brain regions. Red indicates the inhibitory cluster and black indicates the facilitatory cluster (based on the mean ILD function in ***B***). Symbols indicate brain region, squares for AC (and lighter shades) and circles for IC (and darker shades). ***F***, Mean normalized ILD functions of the clusters (where above zero is facilitation and below zero is suppressive). Red indicates inhibitory functions and black facilitatory. ***G***, Percentage of cells falling into each category (facilitatory and inhibitory) in each region.

For each cell, 7 ILD functions were measured: three with a low contralateral frequency (1 × 0 IFD, 2 > 0 |IFD|), three with a high contralateral frequency (1 × 0 IFD, 2 > 0 |IFD|), as described above, and an additional one with a middle contralateral frequency (1 × 0 IFD) for classification purposes. Different types of binaural interaction (excitatory, inhibitory, and numerous variations) have been reported for ILD functions. Here, ILD functions were classified using an objective method. First, ILD functions were normalized by subtracting the contralateral alone response and dividing by the absolute maximum change in firing rate (across all presented conditions). Then, a principle component analysis (PCA) was performed on these data. The Euclidean distance between ILD functions in PCA space (first three components) was used to calculate a dissimilarity matrix. Agglomerative clustering (using Ward's linkage method) was then performed on this matrix to provide a quantitative basis for dividing cells into different classes (see Fig. 2*E*). This resulted in two separate classes: one binaurally inhibitory and one binaurally facilitatory.

**Figure 3.**
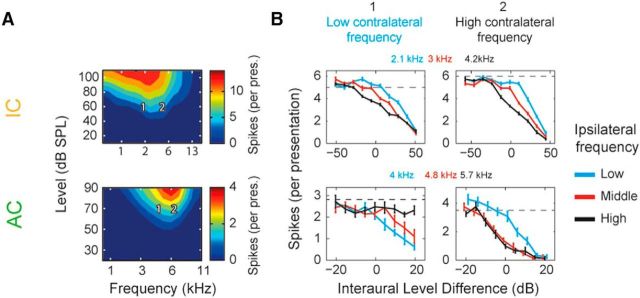
Single-cell examples demonstrating binaurally inhibitory cells compare ILDs over a restricted frequency range in AC (top row), but not in IC (bottom row). ***A***, FRAs of the monaural contralateral ear. Color represents the number of spikes (per presentation) at each frequency (*x*-axis) and level (*y*-axis) combination. Numbers indicate the contralateral frequencies chosen for further investigation. ***B***, ILD functions were collected using a low-frequency (1) and high-frequency (2) contralateral tone and different ipsilateral tone frequencies (low, middle, and high; exact frequencies are shown above panels).

To quantify changes in ILD function produced by changing the IFD, a contrast sensitivity measure was adopted similar to that used to quantify frequency specificity in studies of stimulus-specific adaptation (Ulanovsky et al., 2003). For a given ILD function, we computed the magnitude of the integral over all ILDs, |∑ILD|, of the increase (facilitatory functions) or decrease (inhibitory functions) in the firing rates compared with the responses to the corresponding contralateral tone alone condition. For each extreme contralateral tone condition (low or high), we contrasted this integral when the ipsilateral and contralateral tone frequencies were matched, |∑ILD_IFD__= 0_| with when they were mismatched, |∑ILD_IFD=Δ_|. The interaural frequency specificity index (IFSI) was calculated as follows:

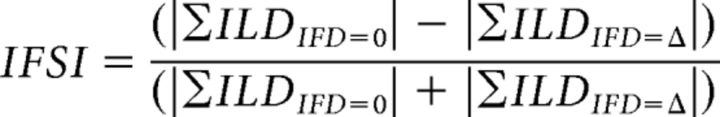
 The IFSI was calculated for both the low-frequency (IFSI_L_) and high-frequency (IFSI_H_) contralateral tones (e.g., see Fig. 3).

In addition, the overall direction of change in firing for a neuron (IFSI_neuron_) was quantified by summing the numerators and denominators for the IFSI_H_ and IFSI_L_ (again, following Ulanovsky et al., 2003) as follows:

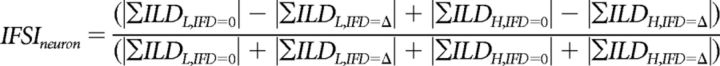
 IFSI_neuron_ was positive if, overall, there was a decrease in the magnitude of inhibition or facilitation when IFD was not zero. Neuron IFSI was negative if, overall, there was an increase in inhibition or facilitation when IFD was not zero. IFSI_neuron_ was zero if no change was observed or if the effect of IFD was of opposing direction for the high- and low-contralateral conditions.

##### Modeling.

A simple firing rate model was used to produce the responses shown in Figure 7, which is intended to demonstrate processing principles rather than being an accurate model of all ILD processing. This model did not include any time dimension; inputs and outputs were assumed to be static during the period of a stimulus. The auditory periphery on each side was modeled as a bank of gammatone filters (Patterson, 1994) implemented in the frequency domain and set to model the bandwidth of guinea pig auditory nerve fibers (Evans et al., 1992). Each pure tone stimulus was defined by a single component in the frequency domain in units of microPascals.

Auditory nerve fibers differ in their threshold and dynamic range (Liberman, 1978) and are integrated centrally in some way which is poorly understood (e.g., Lai et al., 1994). Here, we used a very simple model of transduction that is assumed to represent the result of this integration. Noting that nerve fibers display linear increases in firing rate with sound pressure level within their dynamic range (i.e., dB SPL), the energy at the output of the filters was log transformed. To approximate the threshold of the auditory periphery and to prevent unrealistically low sound levels being represented in the model, a softmax threshold function was applied. For each channel in the filterbank, the output of the peripheral model is as follows:

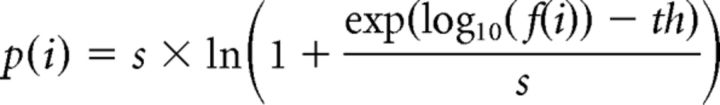
 Where *f*(*i*) is the energy at the output of the filter in channel *i*, *p*(*i*)is the response of the peripheral model, *th* determines the threshold of the model, and the parameter *s* governs the abruptness of the thresholding (for *s* ≪ 1, this approximates a simple threshold i.e., *y* = (*x* − *th*) for *x* > *th*, and *y* = 0 otherwise). The value of *th* was set such that the threshold at the center frequency of each filter corresponded to 20 dB SPL in the model.

Binaural computation in each frequency channel was modeled as simple linear combination of the output from the corresponding filterbank channel from each ear by either adding or subtracting the right (ipsilateral) from the left (contralateral). To model the observation that ipsilateral inhibitory contributions are weaker than the excitatory contribution from the contralateral side (Yu and Young, 2013), inhibitory inputs are weighted by 0.25 as follows:

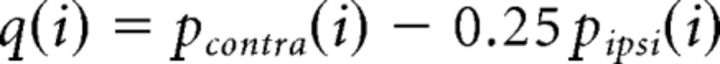
 In the case of a nonlinear model, any negative values were set to zero to model the fact that neurons cannot fire at negative firing rates and therefore cannot pass this information on to downstream neurons. In the case of the linear model, these values were carried forward to the next stage unaltered; that is, unrealistic negative firing rates.

Across-frequency integration of information was modeled as a linear sum of the inputs from the binaural computation stage as follows:

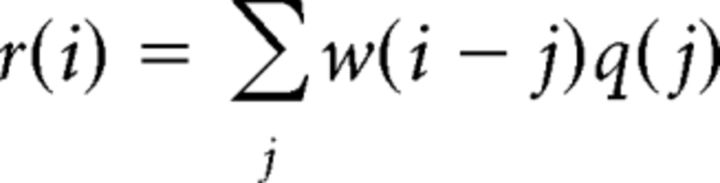
 where *r*(*i*) is the output of a neuron, and *w*(.) is a Gaussian weighting function centered on channel *i*. Therefore, at this level, neuron *i* may have broad-frequency tuning, but will still have a CF corresponding to that of peripheral channel *i*. To model the restricted firing rate of this neuron, any negative values were set to zero and the maximum firing rate was restricted to one (for all models). Responses of the model were calculated for the sets of stimuli, either contralateral pure tones to generate FRAs or the binaural stimulus paradigm exactly as described for the data.

## Results

We assessed the ability of IC and primary AC to make ILD comparisons across different frequency ranges. Pure tones of different frequency and level combinations were presented synchronously to each ear (Fig. 1*A*). The tones in each ear could differ in sound level, creating an ILD across the ears, by fixing the contralateral and varying the ipsilateral sound level (Fig. 1*C*). In addition, the frequency presented to each ear could differ to create an IFD. Analysis of these conditions allowed us to distinguish between the frequency-specific hypothesis and the linear hypothesis.

Recordings were made from cells extracellularly either in primary AC (*n* = 198) or IC (*n* = 118). Cells responded to binaural stimuli with an adapting response that was sensitive to the level of the ipsilateral tones (Fig. 2*A*,*C*). Latency was proportional to the firing rate and there was no evidence that the effect of the ipsilateral tone was delayed relative to the contralateral tone. Therefore, all subsequent data analysis was conducted on the total number of spikes in response to each stimulus (Fig. 2*B*,*D*).

### Cells were classified by clustering their ILD functions

A range of ILD function shapes were encountered in both IC and AC, which is consistent with previous reports (Rutkowski et al., 2000, Nakamoto et al., 2004, Campbell et al., 2006). Qualitatively, it was clear that cells in our dataset exhibited the two main types of binaural interaction described previously: binaural inhibition (EI; Fig. 2*A*,*B*) and binaural facilitation (EE; Fig. 2*C*,*D*). For this reason, we sought to subdivide cells quantitatively based on their spiking responses to different ILDs.

To allow classification of cell type, we first conducted a PCA on the ILD functions from all cells modulated significantly by binaural stimulation (see Materials and Methods). We found that the first three PCs explained >95% of the variance, so these were used for clustering. Figure 2*E* shows the first two PCs of the ILD functions collected. Agglomerative clustering (using Ward's linkage method) revealed two distinct clusters of ILD functions corresponding to EI (red) or EE (black) types. These labels were given to the clusters after inspection of the individual and mean ILD functions (Fig. 2*F*, black and red lines). Both AC and IC cells demonstrate qualitatively similar shapes of individual and mean ILD functions (Fig. 2*E*,*F*). Note that we found no basis for further subdivision of ILD functions in our data and those distinct clusters were not evident if all functions with no significant ILD sensitivity were included in this analysis. Thus the data were consistent with a continuum of binaural interaction types (Campbell et al., 2006).

Multiple ILD functions (seven) were collected for each unit. Most previous descriptions of binaural properties rely on classification of ILD functions where the IFD = 0. Recent work has demonstrated that, in a given cell, ILD functions generally remain of a consistent type (i.e., facilitatory or inhibitory) when frequency is varied but IFD = 0 (Kitzes, 2008). Therefore, our classifications of each cell were based on ILD functions in which the interaural frequency was matched in both ears (i.e., IFD = 0, 3 × ILD functions: contra_L_ipsi_L_, contra_M_ipsi_M_, andcontra_H_ipsi_H_) and a majority rule vote determined the overall classification of a given cell from these ILD functions. Classified this way, we found a large bias toward EI cells in our populations (70% of cortical cells and 81% of IC cells; Fig. 2*G*).

### Binaurally inhibitory cells compare ILDs over a restricted frequency range in AC but not in IC

A total of 163 cells were classified as being binaurally inhibitory and, of these, 91 were cortical and 72 were in IC. Figure 3 shows two example units from the IC and AC. For each cell, a contralateral FRA was collected (Fig. 3*A*) and two contralateral frequency values were then selected at different sound levels to match the contralateral-alone spike counts (Fig. 3*A*, left top and bottom). Figure 3*B* shows the ILD functions collected for the example units, with three IFDs for each contralateral tone frequency as illustrated in Figure 1*C*.

Whether the ILD sensitivity depends on the absolute frequency of the ipsilateral tone or the difference in frequency relative to the contralateral tone (i.e., the IFD) allows us to distinguish two hypotheses. If, in both contralateral tone conditions, the ILD interactions are weaker when |IFD|>0, then ILD processing is frequency specific within a single neuron. This indicates that ILDs are processed in distinct processing channels that are centered on different frequencies. However, if the sensitivity to ILD is largely dependent on the absolute frequency of the ipsilateral tone, then cells behave as if comparing inputs from a single, frequency-tuned, channel from each ear (linear hypothesis).

In AC, we found that binaurally inhibitory cells demonstrated frequency-specific changes to their ILD functions (Fig. 3*B*, bottom left and right). In the example shown, the sensitivity to ILD was greatest when the frequencies were matched across the ears for both contralateral tone frequencies. This is equivalent to the frequency tuning to ipsilateral sound shifting with the contralateral tone frequency. In contrast, IC cells did not demonstrate these changes (Fig. 3*B*, top left and right). This cell demonstrated stronger inhibition of rate for high-frequency ipsilateral tones regardless of the contralateral tone frequency, suggesting a preference in the ipsilateral tuning that is not affected by contralateral tone frequency.

**Figure 4.**
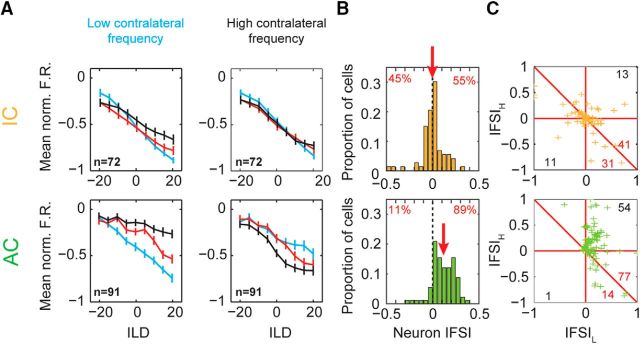
Binaurally inhibitory cells compare ILDs over a restricted frequency range in AC (top row) but not in IC (bottom row) populations. ***A***, Mean population ILD functions for IC and cortical cells (color scheme as in Fig. 3*B*). ***B***, IFSI_neuron_ for IC and AC cells. Positive values indicate reduced suppression and thus a decreased ability to encode ILD. Numbers indicate percentage of population above and below zreo. ***C***, IFSI for IC and AC. Positive values indicate reduced suppression at either the low or high contralateral tone frequency (IFSI_L_ or IFSI_H_, respectively). Number of cells in the bottom left and upper right quadrant are indicated in black. Number of cells above and below the diagonal line are indicated in red.

To confirm this result across our sample of units, we calculated the normalized population mean firing rates for cortical and collicular ILD functions (Fig. 4*A*). In AC, we found large changes in the population ILD functions when a frequency difference was introduced (Fig. 4*A*, bottom). In addition, we found that, at each contralateral frequency, a matching ipsilateral frequency produced the most inhibition, demonstrating the frequency specificity of the interaction. However, in IC, we found that IFD had little systematic effect on ILD functions (Fig. 4*A*, top).

To quantify the amount of frequency specificity in each unit, two metrics were used: the IFSI and IFSI_neuron_ (see Materials and Methods). Both are contrast sensitivity measures that quantify the similarity of the ILD functions in the matched (IFD = 0) and mismatched interaural frequency (|IFD|>0) conditions. For the neuron IFSI (Fig. 4*B*), positive values indicate that, considering both contralateral tone frequencies, there is, on average, a decrease in sensitivity to ILD when interaural frequencies were mismatched, suggesting frequency-specific binaural interactions. In IC, the distribution of IFSI_neuron_ was centered on zero (mean = −0.0026) with a relatively even distribution of values above and below zero (45% and 54%, respectively; Fig. 4*B*). There was no significant difference between the number of cells above and below zero, so there was no evidence for frequency specificity in IC (sign test, *p* = 0.48). In contrast, AC cells were distributed asymmetrically and centered above zero (mean = 0.12, sign test, *p* ≪.01) where the vast majority of values were positive (89% vs 11%). This demonstrated that, across both contralateral tone conditions, cells demonstrated frequency-specific decreases in inhibition when there was a nonzero IFD.

The IFSI quantified this effect at low and high contralateral frequencies separately and these are plotted against one another in Figure 4*C* (IFSI_L_ and IFSI_H_, respectively). Here, a positive value indicates that, for a single contralateral tone condition, inhibition is stronger when the ipsilateral tone frequency matches the contralateral than when it does not. Note that a positive effect of absolute ipsilateral frequency at a single contralateral tone frequency does not by itself indicate frequency specificity. This can arise simply due to normal ipsilateral frequency tuning. However, in the absence of frequency specificity, these effects oppose each other for the two contralateral tone conditions. Therefore, frequency specificity is indicated by points above the descending diagonal line, IFSI_H_ = −IFSI_L_. Quantified in this way, cortical cells were again found to be overwhelmingly frequency specific (Fig. 4*C*, bottom, crosses falling above diagonal = 77 vs below = 14, sign test, *p* ≪ 0.01). In addition, for the majority of cortical cells, IFSI was positive at both contralateral frequencies (Fig. 4*C*, bottom, top right quadrant = 54 vs bottom left = 1), indicating that inhibition was strongest when frequencies were matched across the ears in both contralateral conditions. Conversely, there was little evidence of frequency specificity in IC (Fig. 4*C*, top, crosses falling above diagonal = 41 vs below = 31, sign test, *p* = 0.3). Our data reveal that binaurally inhibitory cells in cortex process ILD cues in a frequency-specific manner not found in IC.

### Larger IFDs produce more interaural frequency specificity in AC

**Figure 5.**
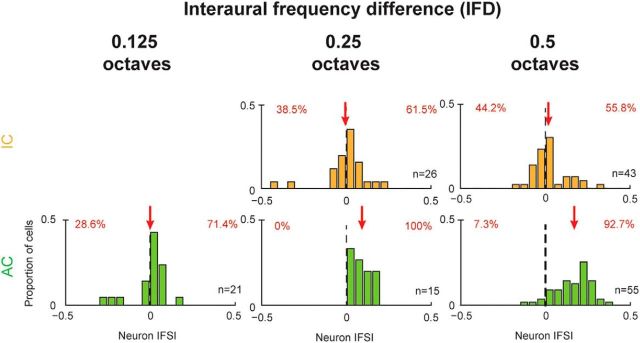
Frequency specificity of binaurally inhibitory cells is dependent on the size of IFD in AC. Even the largest IFDs did not produce frequency-specific effects in IC. Cells in IC and AC (top and bottom rows, respectively) were grouped based on the size of the IFD, where the neuron IFSI was calculated for three IFDs: 0.125 octaves (left), 0.25 octaves (middle), and 0.5 octaves (right). Dashed vertical lines indicate zero IFSI; red arrows indicate the mean IFSI for each subpopulation.

The effect of the size of the IFD on frequency specificity was assessed. Cells were grouped based on the IFD presented and the distribution of IFSI_neuron_ in the IC and AC were compared (Fig. 5). In IC, IFSIs were relatively evenly distributed above and below zero IFSI at all IFDs (where *n* > 2): 0.25 (38.5% vs 61.5%, below and above zero) and 0.5 octaves (44.2% vs 55.8%). A sign test revealed that this was not significant at any of the IFDs included (*p* = 0.33, *p* = 0.54 for 0.25 and 0.5 octave IFD, respectively). In addition, mean IFSIs were also approximately zero for all IFDs (−0.005 and 0.02, respectively), with no significant difference between them (ANOVA, *F* = 0.29, *p* = 0.59). In AC, increasing IFD produced increasing IFSI values. A 0.125 octave IFD produced a slightly positive bias in the distribution of IFSI values (28.6% vs 71.4%, below and above zero IFSI), although this was not significant (sign test, *p* = 0.08). At 0.25 and 0.5 octaves, IFSIs were largely positive (0 vs 100% and 7.3 vs 92.7%) and highly significant (sign test, *p* ≪ 0.01 for both). Increasing IFD values led to larger mean IFSIs (μ = 0.002, 0.09, and 0.17, respectively), demonstrating a significant increase in IFSI with increasing IFD (ANOVA, *F* = 20.26, *p* ≪ 0.01). Overall, the lack of evidence for frequency specificity in IC cells was maintained because even the largest IFDs did not produce a frequency-specific effect. Conversely, AC cells demonstrated increasing frequency specificity with increasing IFD.

### Binaurally facilitatory cells demonstrate no evidence of frequency specificity

**Figure 6.**
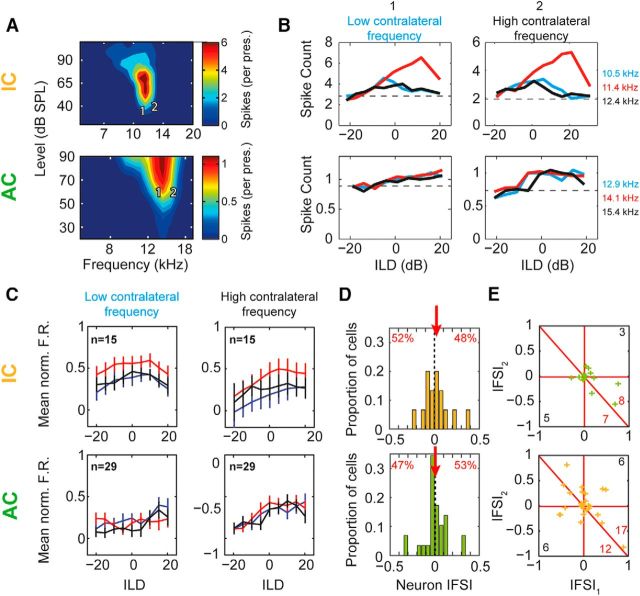
No evidence was found to suggest excitatory cells in either AC or IC demonstrate frequency specificity. ***A***, FRAs of the monaural contralateral ear. Color represents the number of spikes (per presentation) at each frequency (*x*-axis) and level (*y*-axis) combination. Numbers indicate the contralateral frequencies chosen for further investigation. ***B***, ILD functions were collected using a low-frequency (1) and high-frequency (2) contralateral tone and different ipsilateral tone frequencies (low, middle, and high; exact frequencies are shown above panels). ***C***, Mean normalized ILD functions from AC (top row) and IC (bottom row) measured at low (left) and high (right) contralateral frequencies. Line color indicates low, middle, and high ipsilateral tone frequency (blue, red, and black, respectively). ***D***, IFSI_neuron_ for IC and AC cells. Positive values indicate reduced suppression. Numbers indicate percentage of population above and below zero. ***E***, IFSI AC (top) and IC (bottom) cells. Positive values indicate that the matched frequency produced the most suppression; negative values indicate that the opposite frequencies (e.g., ipsi-low, contra-high) produced the most suppression. Red lines mark zero crossings (horizontal and vertical) and where the SI was on average positive or negative (diagonal).

We also recorded from a smaller number (44) of binaurally facilitatory cells (29 in AC and 15 in IC). In contrast to binaurally inhibitory cells, IFD had little effect on ILD functions of facilitatory AC cells; matched-frequency conditions were similar to mismatched conditions (Fig. 6*B*, bottom). Similarly, IC cells demonstrated little change in ILD functions either by changing IFD or contralateral tone frequency (Fig. 6*B*, top). Mean ILD functions were comparable across IFD and contralateral frequency for both AC and IC (Fig. 6*C*). As before, specificity indices were calculated for each cell (Fig. 6*D*,*E*). In both IC and AC, the distributions of neuron IFSIs were centered on 0 (mean = 0.0076 and 0.02, respectively) and were evenly distributed around the mean, with no significant difference (Fig. 6*D*, IC: 52% vs 48%, sign test: *p* = 1, AC: 47% vs 53%, sign test: *p* = 1). Binaurally facilitatory AC cells did not demonstrate evidence of frequency specificity in IFSI (Fig. 6*E*, bottom, above diagonal = 7 vs below = 8, sign test, *p* = 1) and neither did IC cells (Fig. 6*E*, top, above diagonal = 12 vs below = 17, sign test, *p* = 0.45). Overall, we found no evidence for frequency specificity in either AC or IC in binaurally facilitatory cells.

### Conceptual model of across-frequency binaural integration

**Figure 7.**
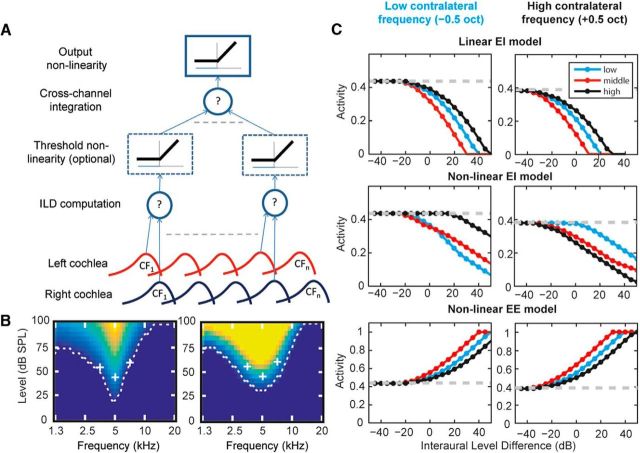
***A***, Linear/nonlinear “constructive-inheritance” model. Cochlea filtering from each ear is added (+) or subtracted (−) in each frequency channel. In a nonlinear model, this passes through a threshold nonlinearity. ILD computations are then linearly summed across frequency channel before a final thresholding output nonlinearity. ***B***, Monaural (contralateral) FRAs from the model at the output from the ILD computation stage (left) and the across-frequency integration stage (right). This response does not depend on the presence of the nonlinearity after ILD computation. ***C***, Example model responses to the binaural stimuli used in the experiment for the following: an EI model with the thresholding nonlinearity omitted, showing no frequency specificity of binaural interaction; an EI model with the thresholding nonlinearity with frequency-specific binaural interactions; and a nonlinear EE model showing no frequency specificity of binaural interaction (see Materials and Methods for model details). The three models differ only in the sign (±) of the binaural computation and the presence or absence of the threshold nonlinearity.

Previous work has suggested that broadband spatial responses are a linear weighted sum of inhibitory and/or excitatory frequency channels from either ear (Schnupp et al., 2001, Mrsic-Flogel et al., 2005). Our results suggest that the integration of level information across ears and across frequency is nonlinear. We implemented a simple model to test how a plausible circuit, and variations in it, might in principle account for our results (Fig. 7*A*). In this model, inputs from the left and right cochlea are first compared, individually for each frequency channel (ILD computation layer). This can be either an excitatory (+) or inhibitory (−) interaction. At a subsequent layer, inputs from these binaurally sensitive neurons are combined (cross-channel integration layer). The model is configured such that the integration can in total be linear, consistent with previous findings, or we introduce a threshold (nonlinearity) before across-frequency integration. Therefore, models can differ in the nature of the binaural interaction (excitatory or inhibitory), and whether they integrate across frequency linearly or nonlinearly.

The outputs for simulations of our experimental paradigm using these different model variations are shown in Figure 7*C*. The linear EI model (Fig. 7*C*, top row), in which the binaural interaction is inhibitory (−) and there is no threshold nonlinearity between layers, does not display frequency specificity; that is, the effect of ipsilateral sound does not depend on the contralateral condition. However, simply adding a threshold nonlinearity between layers (the nonlinear EI model; Fig. 7*C*, middle row) changes the model behavior radically. This nonlinearity creates frequency specificity that is not observed in the linear model. The difference in these models (the nonlinearity) is akin to one model combining frequency and binaural information in the same cells (linear EI) and the other calculating ILD computations in one population of cells and then integrating across frequency in another because subthreshold information is naturally not conveyed between neurons. In contrast, the nonlinearity does not affect processing in the EE model (the nonlinear EE model is shown in Fig. 7*C*, bottom row). Performance is identical with or without this nonlinearity (linear version not shown).

These models suggest that our cortical data are consistent with a nonlinear model in which interactions across the ear, whether excitatory or inhibitory, occur in one population of neurons and that across-frequency integration of binaural information occurs at a later stage. Our IC data, in contrast, are consistent with either a linear model with no clear separation between integration between ears and across frequency or simply a lack of across-frequency integration.

## Discussion

The ability to integrate information across-frequency is important for accurate sound localization. We demonstrate that ILD cues are processed differently at different stages in the ascending auditory system and by different subpopulations of cells.

### ILD processing between the midbrain and cortex

We observed a frequency-specific reduction in binaural inhibition in AC that is not present in the IC. This raises the question: how does cortex demonstrate frequency-specific ILD sensitivity not present in IC?

Our midbrain recordings targeted the central IC, which forms part of the lemniscal pathway projecting to primary AC via the ventral medial geniculate body (vMGB, Shneiderman et al., 1988, Huang and Winer, 2000, Hu, 2003). One possibility is that frequency-specific ILD sensitivity in cortex is inherited from outside the lemniscal pathway. However, AI only receives a minority of its inputs from non-lemniscal divisions of MGB (Huang and Winer, 2000, Hu, 2003, Winer and Schreiner, 2005, Storace et al., 2012) and evidence suggests that cortical ILD sensitivity is inherited from vMGB (Middlebrooks and Zook, 1983, McMullen and de Venecia, 1993) via the CIC (Yao et al., 2015).

A second possibility is that cortical frequency-specific ILD sensitivity is not inherited from the ILD sensitivity in the midbrain. *De novo* creation of ILD functions in MGB is not supported by the anatomical literature (Malmierca et al., 2003) because there is little across-hemisphere interaction (Williams, 1995, Olry and Haines, 2005) and local inhibition via ipsilaterally tuned cells is unlikely due to the paucity of inhibitory interneurons (Winer and Larue, 1996, Winer et al., 1998). In contrast, the corpus callosum provides (disynaptic) inhibitory input via excitatory connections between each cortical hemisphere (Rock and Apicella, 2015). However, recent work has shown that cortical inhibition does not create ILD sensitivity in cortical EI cells (Kyweriga et al., 2014). Therefore, there is insufficient evidence to support significant *de novo* computation of ILDs beyond the midbrain.

A third possibility, suggested by our simple model (Fig. 7), is that frequency specificity arises because binaural interactions precede across-frequency integration. Although frequency tuning in IC neurons varies (Palmer et al., 2013), across-frequency integration is less evident in IC than in cortex (Mc Laughlin et al., 2007, Chen et al., 2012). In contrast, tectothalamic projections into MGB suggest large frequency convergence (Lee and Sherman, 2010) and there is extensive thalamocortical and intracortical convergence of inputs (Miller et al., 2001, Kaur et al., 2005, Winer and Schreiner, 2005, Intskirveli et al., 2016). Therefore, ILD is largely processed in the LSO and IC, whereas frequency integration is more evident in the subsequent projections to MGB and AC, suggesting a partial dissociation of these circuits and in agreement with the model.

Naturally, our model does not represent the full complexity of known ILD computations. In the AC (Kyweriga et al., 2014, Sollini and Chadderton, 2016) and IC (Li and Pollak, 2013, Xiong et al., 2013, Ono and Oliver, 2014), there is ample evidence of local inhibition and excitation shaping responses of all types and in IC and even the cochlear nucleus (Park et al., 1997, Sanes et al., 1998, Burger and Pollak, 2001, Shore et al., 2003, Pecka et al., 2007, Dahmen et al., 2010, Pollak, 2012, Yao et al., 2015, Orton et al., 2016). In addition, recent evidence in the rat suggests sharpening of ILD tuning between IC and MGB (Yao et al., 2015). However, these findings do not explain changes in frequency specificity between IC and AC. The model used here demonstrates how frequency specificity of ILD processing could be a logical consequence of the order with which ILD cues are created and integrated.

### Implications for broadband spatial processing

Unlike our results, Schnupp et al. (2001) found that cortical spatial processing was linear for broadband sounds. Our stimuli were designed to test explicitly for a particular nonlinearity. Model-based characterizations of neural responses typically leave a significant portion of the variance unaccounted for, some of which is due to uncharacterized nonlinearities (Yu and Young, 2013; Machens et al., 2004). This suggests that nonlinear effects are influential, but also that they can be difficult to characterize (Rieke et al., 1999, Bar-Yosef et al., 2002). Our work demonstrates that cortical spatial processing is not linear and highlights the value of testing for specific nonlinearities where they are hypothesized to exist.

It is not certain why the auditory system might process ILD information in a frequency-specific manner, but there are potential advantages. Schnupp et al. (2001) highlight that linear operations have the advantage of preserving information, whereas nonlinear operations select out information, which can be beneficial. The thresholding nonlinearity has the effect of removing information that weakly drives the network; in the case of sound localization in cortex, ipsilateral sounds. Take the example of two competing sounds: a target (broadband/contralateral) and a masker (high-frequency/ipsilateral). In the nonlinear model, the effect of the masker will be limited by the additional threshold and, after frequency integration, a neuron would reflect only those frequencies dominated by the contralateral signal. In contrast, a linear system will still encode both sound sources after across-frequency integration despite the spatial separation. Therefore, the mechanisms that likely lead to frequency specificity could influence the robustness of representation for multiple sources (Keller and Takahashi, 2005) and thus affect the detectability and localizability of sounds in noisy environments.

Another potential advantage of processing ILD in a frequency-specific manner is in the straightforward weighting of cues. ILD cues are frequency specific, the size of the ILD varies with frequency, and, due to the Head Related Transfer Function, often in a nonmonotonic manner (Shaw, 1974, Sabin et al., 2005). ILDs being computed first in narrow-frequency bands means that they can be weighted separately, allowing the most useful frequency ranges regions to be strongly weighted and the weakest to be weakly weighted before being synthesized.

The fact that EI cells have broad hemispheric tuning (Nakamoto et al., 2004, Zhang et al., 2004) suggests that a straightforward rate code of ILD is insufficiently precise to underlie the ∼1 dB ILD discrimination thresholds observed in humans (Colburn and Durlach, 1978). This has led to the suggestion that opponent (hemispheric) channels could explain high-frequency sound localization behavior (Stecker et al., 2005). This implies EI cells are more suited to computing sound location because they demonstrate hemispheric tuning, whereas EE cells are tuned to the midline (Rajan et al., 1990, Zhang et al., 2004). Our data further support this because we found that EI cells process ILD in a frequency-specific manner consistent with human behavior (Francart and Wouters, 2007, Goupell et al., 2013), whereas EE cells do not.

### Impact of frequency-specific processing on spatial perception

Across-frequency integration is clearly important for sound localization. Increasing sound bandwidth improves sound localization performance by adding information and resolving ambiguities in narrowband localization cues (Terhune, 1974, Butler, 1986, Wightman and Kistler, 1992). Our results imply that cortical EI processing allows the necessary combination of cues while limiting interactions of binaural cues between different frequency regions that could otherwise lead to errors in ILD computation. Qualitatively consistent with this, perceptual sensitivity to ILD decreases with increasing IFD (Francart and Wouters, 2007, Goupell et al., 2013). In addition, it suggests that, for bilateral cochlear implants, a physiological constraint exists: stimulating electrodes need to be well matched for position/frequency to yield good ILD lateralization, again consistent with the literature (Kan et al., 2013).

### Generalizability of frequency specificity

Frequency specificity of binaural interaction bears a striking similarity to frequency-specific adaptation (more generally, stimulus-specific adaptation, SSA), which has been reported in the IC (Malmierca et al., 2009, Duque et al., 2012, Ayala et al., 2015), thalamus (Anderson et al., 2009, Antunes et al., 2010), and AC (Ulanovsky et al., 2003, 2004, von der Behrens et al., 2009, Scholes et al., 2011).

SSA generally increases in strength along the auditory pathway and there is variation between subdivisions at each stage. In the IC and thalamus, it is largely restricted to non-lemniscal regions (Anderson et al., 2009, Antunes et al., 2010, Ayala et al., 2015) and SSA is strongest in the AC (Nieto-Diego and Malmierca, 2016) in non-lemniscal regions. Although we only targeted lemniscal regions of IC and AC, it is reasonable to hypothesize that more broadly tuned non-lemniscal regions of IC and AC, which also tend to show improved spatial acuity (Recanzone, 2000, Harrington et al., 2008), might show strong frequency-specific binaural interactions.

The findings in SSA may also offer clues to the underlying mechanisms of binaural frequency specificity. Many (though not all: Taaseh et al., 2011) of these data are also consistent with a constructive inheritance model (Miller et al., 2001, Winer and Schreiner, 2005). Both our data and SSA can be explained by a convergence of information with either comparisons across the ears or adaptation occurring independently within separate frequency-specific channels (Mill et al., 2011, Duque et al., 2016). Manipulations of GABAergic, glutamatergic, and cholinergic synapses (Ayala et al., 2016) and descending control from AC (Malmierca et al., 2015) also suggest that SSA in IC is influenced by changes in overall excitability or adaptation rather than being crucially dependent on them, again supporting the model of constructive convergence. Other similarities between the phenomena include larger effects with increased frequency difference (Ulanovsky et al., 2003; cf. Fig. 5) and a stronger effect above CF than below it (Duque et al., 2012; cf. Fig. 4). Therefore, there is evidence across disparate phenomena for this form of integration, which may be a common architectural feature in the auditory pathway (Williamson et al., 2016).

### Summary

We have demonstrated that the cortical integration of ILD cues across frequency nevertheless preserves the frequency-specific nature of the physical cue. This frequency specificity is similar to stimulus-specific adaptation in its action and the location where the specificity develops. It places distinct constraints on the way that the auditory system processes location and suggests that nonlinear, within-channel computation of interaural level cues subcortically precedes a process of constructive integration in primary AC or thalamus.
